# Public Perceptions of Environmental Public Health Risks in the United States

**DOI:** 10.3390/ijerph16061045

**Published:** 2019-03-22

**Authors:** Mikyong Shin, Angela K. Werner, Heather Strosnider, Lisa B. Hines, Lina Balluz, Fuyuen Y. Yip

**Affiliations:** 1 Environmental Public Health Tracking Section, Division of Environmental Health Science and Practice, National Center for Environmental Health, Centers for Disease Control and Prevention, Atlanta, GA 30341, USA; awerner@cdc.gov (A.K.W.); hstrosnider@cdc.gov (H.S.); fyip@cdc.gov (F.Y.Y.); 2 ORISE Postdoctoral Fellow at the Environmental Public Health Tracking Branch, National Center for Environmental Health, Centers for Disease Control and Prevention, Atlanta, GA 30341, USA; 3 National Center for Injury Prevention and Control, Centers for Disease Control and Prevention, Atlanta, GA 30341, USA; lhines@cdc.gov; 4 Division of Toxicology and Human Health Sciences, National Center for Environmental Health, Centers for Disease Control and Prevention, Atlanta, GA 30341, USA; lballuz@cdc.gov

**Keywords:** audience segmentation, awareness, concern, *ConsumerStyles*, environmental health, government, risk communication, risk perception, survey, tracking

## Abstract

Understanding public perceptions about environmental health hazards, exposures, and health impacts can help environmental public health practitioners to target and prioritize community activities, policy needs, and communication strategies. The online cross-sectional 2013 summer wave of the *ConsumerStyles* survey sampled U.S. adults and used questions from the Centers for Disease Control’s Environmental Public Health Tracking Program to measure public awareness of governmental efforts to track environmental exposures and links to health impacts, as well as perceptions of environmental health issues. Unadjusted and adjusted logistic regressions examined the associations between demographic characteristics and level of awareness of government environmental public health efforts or level of concern about health risks associated with environmental pollutants. Responses were received from 4033 participants, yielding a response rate of 66.0%. More than half of respondents (57.8%) noted concerns about health risks from environmental pollutants. More than one-third (40.0%) of respondents reported awareness of government efforts. Nearly 40% of respondents felt that none of the health impacts listed in the survey were related to environmental issues. Multiple logistic regression models showed that non-Hispanic blacks, other races, females, people with a college or higher education, and people living in the Midwest or South regions were more likely than their counterparts to be concerned about how the environment affects their health. Future work should focus on improving risk communication, filling the information gap on environmental health issues, and understanding how perceptions change over time.

## 1. Introduction

Risk perception refers to a person’s beliefs about their vulnerability to harm and the severity of a hazard [1,2]. This is an important concept in public health as it influences the hazards that people are concerned about and how people deal with those hazards [3]. Generally, the public evaluates risks based more on their subjective perceptions and intuition as well as inferences from a limited set of information, including media coverage, and less on knowledge of objective risk factors [3].

Specific to environmental health risks, a survey of registered voters conducted in 2000 showed that most respondents believed environmental problems posed a risk to health and were worried about exposures to environmental conditions that may have health impacts [4]. Many respondents thought a monitoring system already existed to track environmental hazards and their links to chronic illness. When learning that no such system existed at that time, 84% of respondents expressed concern, with a majority of respondents noting that establishing a monitoring and tracking network would be one of the most important things the government could do [4]. In that same year, the Pew Environmental Health Commission stated that America’s environmental public health system was fragmented, neglected, and ineffective, calling for the creation of an environmental public health tracking network [5,6]. In response to this call for action, in 2002, the Centers for Disease Control and Prevention (CDC) established the National Environmental Public Health Tracking Program (Tracking Program) to bridge these existing data gaps and track exposures and health effects associated with environmental hazards [5].

The Tracking Program’s Tracking Network provides data on health effects, environmental hazards, and exposures. Indicators and measures that are displayed on the Tracking Network are developed based on CDC priorities, state and local health departments, environmental health experts, and public opinions and ideas.

Now that a formal government system has been established and has served to track environmental hazards and links to chronic illness for over one decade, the Tracking Program decided to survey public perceptions on awareness of governmental efforts to track these issues and concerns about health risks from the environment and environmental health issues. The findings will allow the Tracking Program to refine and focus its communication messages and outreach strategies to reach groups who may be interested in using the Tracking Network. The findings can also help the Tracking Program prioritize future content. The work will help to better understand how to educate the general public, health care professionals, the media, and others on environmental public health issues.

## 2. Materials and Methods

### 2.1. Data Source

Porter Novelli regularly administers the *ConsumerStyles* survey, a series of online-based surveys that aim to gather insights about American consumers, measuring health knowledge, attitudes, and behaviors of U.S. adults [7].

### 2.2. Survey Panel and Participants

In Spring 2013, Porter Novelli sent the *ConsumerStyles* survey to a national random sample of 7988 adults aged 18 years or older who belonged to KnowledgePanel^®^ [8] whose members are randomly recruited using online survey samples, using probability-based sampling. The panel is replenished regularly, maintaining approximately 55,000 panelists [8] regardless of landline phone or Internet accessibility.

Adults who completed the spring wave of the *ConsumerStyles* survey (hereafter referred to as SpringStyles) were emailed the summer wave of the *ConsumerStyles* survey (i.e., SummerStyles) in June/July 2013. The SummerStyles survey was completed by 4033 (66.0%) of 6102 SpringStyles respondents. The median completion time was 18 min. Respondents were not required to answer any of the questions and could exit the survey at any time. Respondents who did not answer at least half of the questions were classified as incomplete and removed from the dataset. Respondents who completed the survey received reward points (worth approximately $5) and were entered into a monthly sweepstakes.

Porter Novelli weighted the data to match the U.S. Current Population Survey proportions for age, census region, education level, household income, household size, internet access prior to joining the panel, metro status, race/ethnicity, and sex [7]. Personal identifiers were not included in the data file; therefore, analyses of these data were exempt from institutional review board approval.

### 2.3. Survey Questions

The Tracking Program developed five survey questions that were included in the 2013 SummerStyles survey related to awareness of governmental efforts to track environmental hazards and links to health problems and perceptions of environmental health issues. The questions were developed by communication specialists and scientists in the Tracking Program along with *ConsumerStyles* survey experts based on information about people’s environmental health beliefs and concerns found in existing literature, gaps in the literature, and goals of the Tracking Program. The format of the questions and answers were also guided by the *ConsumerStyles* survey format. The questions and available responses for each question are shown in Supplement Table S1.

### 2.4. Outcome Variables

#### 2.4.1. Level of Awareness

Respondents were asked about their awareness of government efforts to track environmental hazards and possible links to chronic health problems. Respondents rated the statement “I am aware of the government’s efforts to track environmental hazards and possible links to chronic health problems” (shown in Table S1), with the choices of strongly disagree, somewhat disagree, neither agree nor disagree, somewhat agree, and strongly agree. Three categories were created for the level of awareness variable: aware, neutral, and not aware. The aware group comprised the “strongly agree” and “somewhat agree” responses, the not aware group was composed of the “strongly disagree” and “somewhat disagree” responses, and the neutral group included the “neither disagree nor agree” responses.

#### 2.4.2. Level of Concern

Respondents were asked about their level of concern, responding to the following statement: “How concerned are you about the risks to your health from pollutants in the environment?” (question 2 shown in Table S1). Answer choices were not at all concerned, not very concerned, somewhat concerned, and very concerned. Respondents were categorized into two groups: concerned and not concerned. The concerned group included the “very concerned” and “somewhat concerned” responses, and the not concerned group comprised the “not at all concerned” and “not very concerned” responses.

### 2.5. Sociodemographic Characteristics

Sociodemographic characteristics included sex (male or female), race/ethnicity (non-Hispanic white, non-Hispanic black, Hispanic, or non-Hispanic other (other race and two more races)), age (18–34, 35–64, or 65+ years), education (≤high school graduate, some college, or ≥college graduate), marital status (married, partnership, or other), employment status (employed, not employed, or retired), household income (≤$34,999, $35,000–75,000, or >$75,000), household size (1, 2, 3, 4, or 5 or more people), and home ownership status (yes or no). Chi-square tests and univariate logistic regression were used for sub-groups to test for any differences in characteristics. Respondents were classified according to U.S. Census regions (Northeast, Midwest, West, or South), and were asked to rate their health status (excellent, very good, good, fair, poor).

### 2.6. Data Analyses

Chi-square tests were used to examine differences between the sub-groups for the outcome variables. This included an assessment of differences between those in the “aware”, “neutral”, and “not aware” groups and a separate assessment of the “concerned” and “not concerned” groups. The most frequently chosen environmental issues, health conditions, and sources of information were ranked. The two dependent variables for regression models were (1) level of awareness about government efforts to track environmental hazards and possible links to chronic health problems and (2) level of concern about the risk to respondents’ health from environmental pollutants. Respondents (*n* = 44) who did not respond to both outcome variables (i.e., awareness and concern) were removed prior to logistic regression analyses.

For the level of awareness, two univariate logistic regression models were used to estimate odds ratios (ORs) for the “aware” group versus the “not aware” group and the “neutral” group versus the “not aware” group. Results were similar between models; therefore, only the outcome variable comparing the “aware” to the “not aware” group was used in the final multiple regression analysis. For the level of concern related to health risks, one univariate logistic regression model was used to estimate ORs for the “concerned” group versus the “not concerned” group. Multiple logistic regression models were used to compare the “aware” group to the “not aware” group and the “concerned” group to the “not concerned” group whilst adjusting for key sociodemographic factors. Adjusted ORs (aORs) with 95% confidence intervals (CIs) were reported. SAS version 9.3 was used for analyses.

## 3. Results

The majority of respondents were older and non-Hispanic white, with a high school education or less. Over half of respondents rated their health as good, fair, or poor (Supplementary Table S2).

**Figure 1:**
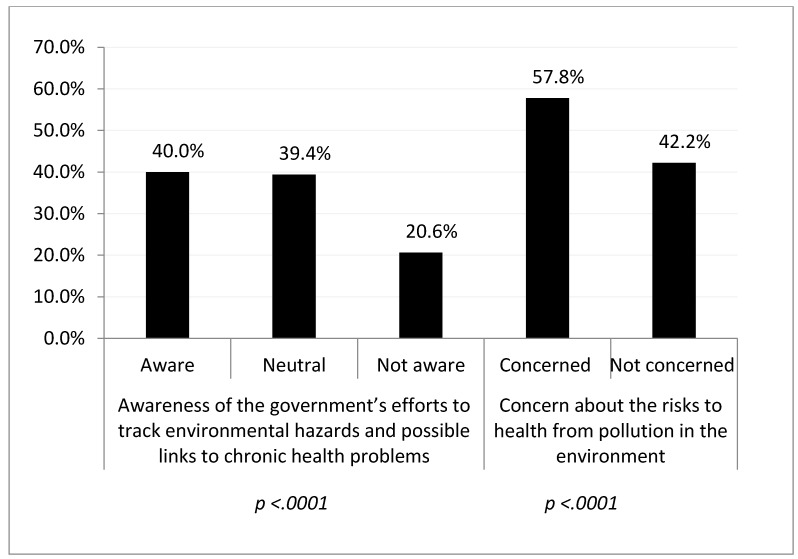
Responses for the two outcome variables (i.e., level of awareness and concern), 2013 SummerStyles. Note, *p* < 0.0001 for differences between sub-groups for both outcome variables.

Of the 4033 respondents analyzed, over one-third strongly or moderately agreed (40.0%) that they were aware of the government’s efforts to track environmental hazards and possible links to chronic health problems. More than half of the respondents reported being very or somewhat (57.8%) concerned about the risk to their health from environmental pollutants (Figure 1). There were significant differences between the subgroups for both outcome variables (*p* < 0.0001).

**Figure 2:**
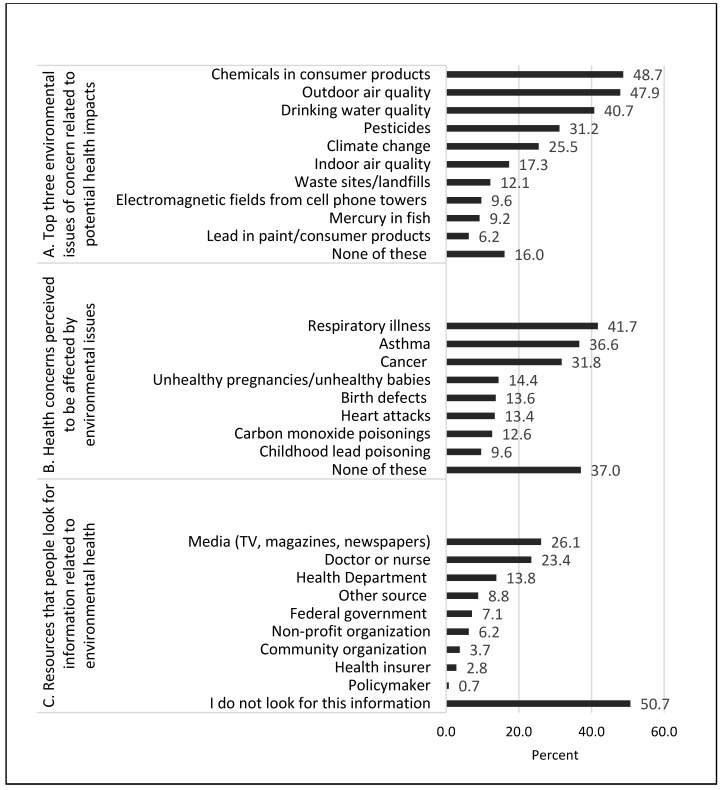
The (**a**) top environmental issues of concern related to health issues, (**b**) health concerns perceived to be related to environmental issues, and (**c**) resources where respondents reported receiving their environmental health information, from 2013 SummerStyles.

Respondents chose chemicals in consumer products (48.7%), outdoor air quality (47.9%), and drinking water quality (40.7%) as the top three environmental issues of concern that may affect their health. These top three choices were followed by pesticides (31.2%), climate change (25.5%), and indoor air quality (17.3%), as shown in Figure 2A. Respondents chose respiratory illness (41.7%), asthma (36.6%), and cancer (31.8%) as the health issues they think may be affected by the environment in their communities. More than 10% of respondents reported unhealthy pregnancies/unhealthy babies (14.4%), birth defects (13.6%), heart attacks (13.4%), and carbon monoxide poisonings (12.6%) as health issues affected by the environment in their communities (Figure 2B). However, nearly 40% of adults reported that they do not think any of the health outcomes are affected by the environment in their communities. Respondents reported turning to the media (i.e., TV, magazines, newspapers) (26.1%), doctors or nurses (23.4%), and the health department (13.8%) as the main resources for obtaining environmental health information (Figure 2C). Only 0.7% of respondents reported turning to a policymaker for information, and approximately half of respondents (50.7%) reported that they do not look for environmental health information.

Univariate logistic regression models examined each demographic factor and both the level of awareness of the government’s efforts to track environmental hazards and possible links to chronic health problems and the level of concern about health risks from environmental pollutants. In comparing the “aware” and “not aware” groups, those over 65 years old were more likely to be aware of governmental efforts to track environmental hazards (OR: 2.07, 95% CI: 1.47–2.90), as were males (OR: 1.50, 95% CI: 1.16–1.92). For the “aware” and “not aware” comparisons, age, sex, education, health status, employment, and income were significant, while the “neutral” and “not aware” comparisons showed race/ethnicity, sex, and health status as significant. The “aware” versus “not aware” and “neutral” versus “not aware” groups showed similar trends between models. In comparing the “concerned” and “not concerned” groups, all race/ethnicities were more concerned about the risks to health from pollutants in the environment compared to non-Hispanic whites (OR: 1.69, 95% CI: 1.21–2.36 for non-Hispanic blacks; OR: 1.38, 95% CI: 1.02–1.88 for Hispanic; and OR: 1.89, 95% CI: 1.22–2.95 for other). Other significant factors included sex, health status, and region. All univariate logistic regression model results are shown in Supplementary Table S3.

The multiple logistic regression model using the awareness outcome variable included the following factors of interest, based on the univariate models: age, sex, education, and health status. Table 1 shows the results from the multiple logistic regression model examining awareness of government efforts to track environmental hazards and links to chronic health problems. In these models, respondents in the middle age group (35–64 years old) and oldest age group (≥65 years old) were more likely than younger respondents to be aware of governmental efforts to track environmental hazards and possible links to chronic health problems (aOR: 1.54, 95% CI: 1.16–2.06 and aOR: 2.49, 95% CI: 1.76–3.54, respectively). Respondents who had some college education or who were college graduates or higher were 47% and 107% more likely to be aware of the government’s efforts compared to respondents who were high school graduates or less (aOR: 1.47, 95% CI: 1.08–1.99 and aOR: 2.07, 95% CI: 1.51–2.84, respectively). Females were 39% less likely to be aware of these efforts compared to males, and those who reported fair, or poor health status were 31% less likely to be aware of these efforts compared to those reporting excellent or very good health status.

The multiple logistic regression model using the concern outcome variable included the following factors of interest, based on the univariate models: Race/ethnicity, sex, education, health status, and region. Table 2 shows the results from the multiple logistic regression model examining concerns about risks to health from pollutants in the environment. Non-Hispanic black respondents were 64% more likely than non-Hispanic whites to be concerned about health risks from pollutants in the environment (aOR: 1.64, 95% CI: 1.17–2.31), and other races were 67% more likely to be concerned compared to non-Hispanic whites (aOR: 1.67, 95% CI: 1.07–2.60). Females were more likely to be concerned about health risks compared to males (aOR: 1.50, 95% CI: 1.24–1.81) as were those who reported being college graduates or higher compared to those who reported being high school graduates or less (aOR: 1.30, 95% CI: 1.03–1.63). Respondents who self-reported their health status as fair or poor and good were 36% and 26% more likely, respectively, to be concerned about health risks due to pollutants in the environment compared to those who self-reported their health status as excellent or very good. Finally, respondents in the West region were 44% more likely to be concerned about health risks from pollutants in the environment compared to respondents in the Midwest region (aOR: 1.44, 95% CI: 1.08–1.92, respectively).

## 4. Discussion

This study investigated levels of awareness on government efforts to track environmental exposures and potential health effects and levels of concern related to environmental public health issues. The results showed that the majority of respondents were concerned about risks to health from pollution in the environment, and approximately 20% of respondents were unaware of governmental efforts in tracking hazards and possible links to chronic health issues. Notably, nearly 40% of respondents indicated that they felt none of the health effects are affected by the environment in their communities and half of the respondents reported not looking for information related to environmental health.

The health issues listed in the survey, such as asthma, cancer, and childhood lead poisoning, have clear links to environmental issues. Epidemiological studies have demonstrated increased risk for cardiovascular events related to short- and long-term exposure to ambient particulate matter [9,10,11,12], yet only 13.4% of respondents identified heart attacks as a health outcome affected by the environment. Because such a high proportion of respondents stated that none of the health effects are related to the environment, it is important for the Tracking Program to communicate these links more clearly and disseminate these messages to a broader audience so that the general public can develop a better understanding that these health effects are related to the environment. This is an important information gap to address.

Nearly half of the respondents reported chemicals in consumer products (e.g., phthalates, BPA) and outdoor air quality as top environmental concerns, followed by drinking water quality. A survey of health care professionals and parents and guardians showed that both groups ranked environmental tobacco smoke and pesticides in water as the issues of greatest concern [13]. Another survey found that many parents were concerned about outdoor air quality but did not report as much concern about indoor air quality [14]. This is similar to our study, where concerns about outdoor air quality (47.9%) were much greater than indoor air quality (17.3%) even though research suggests that indoor air quality could be a greater health hazard than outdoor air quality due to factors like tobacco smoke, volatile organic compounds, cleaning materials, and poor ventilation [14]. Finally, the environmental health survey conducted in 2000 showed respondents viewed toxic waste as the top environmental health risk, followed by drinking water with harmful chemicals, water pollution, and pesticides in food [4]. This may reflect changing views and knowledge on environmental health issues over a 13-year period.

Nearly all risk perception studies have shown that men are less concerned about hazards than are women, with women having higher levels of risk perception [15,16]. People with children, particularly women, have also been found to show concern about environmental impacts on their health and implement behavior changes because of these perceived impacts [17,18]. Pregnant women and new mothers are often the key audience for media and public health campaigns [19]. Our results show that the Tracking Program may want to conduct more outreach to this segment of the population to increase awareness.

It is known that environmental risks and the perceptions of those risks are unevenly distributed across different groups in society [20]. In this study, respondents in some minority groups reported greater concern about risks to health than their non-Hispanic white counterparts. It is likely that complex sociopolitical and cultural factors account for these differences. Other work confirmed that non-white respondents tend to be more concerned about environmental issues compared to white respondents [21]. Studies have shown that there are disparities in health conditions related to air pollution exposures [22,23] and that there are significant racial and socioeconomic disparities in the distribution of hazardous waste facilities [24]. There is evidence that minority populations face greater environmental risks, which likely contributes to greater concern about those risks.

Our study found no statistically significant associations between reported level of concern and age group, however, younger respondents in our study showed significantly less awareness about governmental efforts compared to their older counterparts. Study results are mixed on the effect of age on environmental public health risk perception [16]. One study found that there was greater concern about pollution and associated health impacts amongst younger age groups [25]. It was posited that this was because younger people likely had more exposure to these issues at school and because of their culture [25]. However, another study’s findings contradict this, noting that older people in the U.S. have higher levels of environmental concern compared to younger adults [21].

Over one quarter of respondents reported turning to the media as a source of environmental health information. This is not surprising, given that the media is the most common source of environmental risk information [14]. The media can influence risk perception in several ways. Media sources and perceived trustworthiness, level of coverage, media types, and presentation format all factor in to how someone perceives risks that are presented [3]. Likewise, risk perception may change depending on how the information is presented (e.g., noting the average reduction in life expectancy for exposure to a certain hazard versus the number of additional deaths per year due to the same exposure) [15]. The question in this survey grouped all media into one response category, which limits clarification of the different types of media that respondents may use to obtain environmental public health information. Future survey questions should consider the different types of media and the influence on risk perception, including how social media influences environmental public health risk perception. Additionally, the media needs to be educated about environmental public health issues, which can help improve the public’s general understanding of these issues [14].

All of this is also important to consider for risk communication. Risk communication is a “complex, multidimensional, evolving approach to communicating with the public about issues that pose a threat to health, safety, or the environment” [2]. Some public health communicators remarked that members of the general public tend to be savvier about environmental health issues than they were in the past [26]. However, this is still likely to include only those who are interested in, and looking for, this type of information. The results from our study showed that just over half of respondents reported not looking for this information at all. To be effective, risk communicators must acknowledge the myriad of factors that contribute to an individual’s risk perception and they must convey messages in light of numerous challenges, such as lack of trust and innumeracy [26]. Oftentimes, poor communication contributes to higher levels of anxiety, escalating concerns about certain risks, and polarization between different groups [27,28]. Nearly one quarter of respondents reported turning to health care professionals for environmental health information. This should be acknowledged because physicians and other health care professionals play an important role in communicating environmental health risks [29], so it is important that they understand key risk communication principles.

This study has several limitations. The survey was a cross-sectional survey that relied on self-reported data from a convenience sample. Results from this survey are not necessarily generalizable to the U.S. population, although the data were weighted to more accurately reflect the U.S. population using nine factors: age, household income, race/ethnicity, household size, education, census region, metro status, and prior Internet access. The sample population drawn for this survey was from a cohort of people who agreed to participate in market research, making it subject to selection bias as people who participate in such studies may have different characteristics than those who do not. The survey response rate was good (66%), but differences between respondents and non-respondents within the cohort could contribute to biased results due to non-response bias. Additionally, the sociodemographic or residential categories that were used in this study could possibly group dissimilar people. While this survey brings insights about a respondent’s levels of concerns and awareness, the addition of qualitative methods would allow for deeper insight and allow gaps to be filled, further informing environmental risk perceptions [17,30].

The survey questions focused on which environmental issues impact health as well as health issues affected by the environment specific to a respondent’s own community. A respondent’s answers to these questions do not necessarily translate to general environmental public health knowledge, as responses to these questions may be different if they were asked about top environmental issues that impact health or health issues that may be affected by the environment in general. There are also aspects to consider related to the timing of the survey. Research has shown that environmental concern is cyclical, with levels of concern about the environment fluctuating depending on other issues like the economy and war [31]. Based on this, one could presume that, based on the questions used in this survey, reported levels of environmental concern will fluctuate depending on the leading domestic issues. The results could vary, depending on other factors, if this survey were to be conducted at another point in time or included the consideration of other geopolitical factors.

Over time, understanding environmental public health impacts like those discussed in this paper has led to actions such as removing lead from paint and gasoline, placing carbon monoxide detectors in homes, and providing local air quality health alerts [32,33,34]. The CDC’s Tracking Program is using the results from this survey to better understand people’s awareness of governmental efforts and concern about environmental public health issues to identify gaps, consider additional content areas for the Tracking Network, work on educating the media and health care professionals, and improve communication messages to key segments of the public. In response to these results, the Tracking Program worked with Porter Novelli to build two audience profiles for communication and outreach. The profiles were used to develop new communication tools such as infographics, animated maps, social media content, videos, and an online advertising campaign to expand communication channels to any health professionals. These tools address some of the knowledge gaps identified by the survey respondents, particularly the relationship between outdoor air quality and heart disease. Although the Tracking Network does not currently have any data related to chemicals in consumer products, this was a top concern for half of the survey respondents. This is something for the Tracking Program to consider in the future.

Respondents’ lack of connection between their environmental health concerns and their own communities served as a driving force behind the redesigned “Info by Location” search function, which has been available on the Tracking Network since October 2014. This new search function allows users to input ZIP code or county location and receive common environmental and health data for that location in an easily understandable format. Dissemination of these results will help other public health professionals assess people’s awareness and concerns about environmental health and inform actions and strategies for advising people on protecting their health against environmental health risks.

The Tracking Program can consider incorporating these questions into future *ConsumerStyles* surveys to track how a person’s perceptions change over time in relation to their community as more strategies are incorporated for increasing awareness and addressing environmental public health concerns. Additional questions or future studies should delve deeper to better understand the specific environmental public health issues that are of concern to different subpopulations. More work is needed to understand not only how but why different groups perceive environmental risks differently and the implications of this [20]. Future communications work should target how to reach the respondents who reported not looking for any environmental public health information, and focus on providing environmental health training and informative educational resources [35] for health care professionals so these key audience segments can be reached.

Another point that the Tracking Program needs to consider is using information to understand and assess its impact and outreach efforts since its inception. While the Tracking Program does not routinely rely on traditional media sources, it does use social media to provide science-based environmental health information. Expanded outreach through traditional media sources could improve the program’s ability to reach different audiences and raise awareness of these issues. One way that the Tracking Program evaluates its impact is through public health actions, which allows Tracking Program recipients to highlight the work that they are doing. Program outreach efforts are assessed through social media and web metrics as well as a variety of process measures after in-person events. It is possible that improving these outreach efforts to increase reach and gain visibility will add to the level of awareness of these governmental efforts.

## 5. Conclusions

This cross-sectional study provided insight on public perceptions of awareness of government efforts to track environmental hazards and their links to chronic illness and concern about health risks from environmental issues. The CDC’s Tracking Program is using these results to identify key environmental public health information gaps, add additional content areas to the Tracking Network, and improve communication messages to certain audiences. Future work should focus on how these perceptions change over time as the Tracking Program expands its outreach and messaging efforts. Additional work should focus on educating health care professionals and the media to improve public awareness of environmental public health issues.

## Figures and Tables

**Table 1:** Multiple logistic regression: Demographic characteristics by awareness of governmental efforts on environmental health, United States, 2013 SummerStyles.

SummerStyles© Characteristics	Awareness of the Government’s Efforts to Track Environmental Hazards and Possible Links to Chronic Health Problems
	Adjusted odds ratios (95% CI)
	Aware vs. not aware ^1^
Age (years)	
18–34	Ref
35–64	1.54 (1.16–2.06) **
65+	2.49 (1.76–3.54) ***
Sex	
Male	Ref
Female	0.61 (0.47–0.79) ***
Education	
High school graduate or less	Ref
Some college	1.47 (1.08–1.99) *
College graduate or higher	2.07 (1.51–2.84) ***
Health status	
Excellent, very good	Ref
Good	0.74 (0.50–1.08)
Fair, poor	0.69 (0.52–0.91) **

^1^ Aware = “strongly agree” and “somewhat agree” responses; not aware = “strongly disagree” and “somewhat disagree” responses; and neutral = “neither disagree nor agree” responses. *n* = 2404. Notes: * *p* < 0.05, ** *p* < 0.01, *** *p* < 0.001.

**Table 2:** Multiple logistic regression: Demographic characteristics by concerns about health risks from environmental pollutants, United States, 2013 SummerStyles.

SummerStyles© Characteristics	Concern about the Risks to Health from Pollutants in the Environment
	Adjusted odds ratios (95% CI)
	Concerned vs. not concerned ^1^
Race/ethnicity	
Non-Hispanic white	Ref
Non-Hispanic black	1.64 (1.17–2.31) **
Hispanic	1.34 (0.98–1.85)
Non-Hispanic Other ^2^	1.67 (1.07–2.60) *
Sex	
Male	Ref
Female	1.50 (1.24–1.81) ***
Education	
High school graduate or less	Ref
Some college	1.03 (0.82–1.30)
College graduate or higher	1.30 (1.03–1.63) *
Health status	
Excellent, very good	Ref
Good	1.26 (1.03–1.55) *
Fair, poor	1.36 (1.02–1.82) *
Region ^3^	
Midwest	Ref
Northeast	1.26 (0.94–1.69)
South	1.28 (.998–1.65)
West	1.44 (1.08–1.92) *

^1^ Concerned = “very concerned” and “somewhat concerned” responses and not concerned = “not at all concerned” and “not very concerned” responses. ^2^ Other race/ethnicity = Non-Hispanic other races or two more races. ^3^ Northeast includes: Connecticut, Maine, Massachusetts, New Hampshire, Rhode Island, Vermont, New Jersey, New York, and Pennsylvania. Midwest includes: Illinois, Indiana, Iowa, Kansas, Michigan, Minnesota, Missouri, Nebraska, North Dakota, Ohio, South Dakota, and Wisconsin. South includes: Alabama, Arkansas, Delaware, Florida, Georgia, Kentucky, Louisiana, Maryland, Mississippi, North Carolina, Oklahoma, South Carolina, Tennessee, Texas, Virginia, Washington, D.C., and West Virginia. West includes: Arizona, California, Colorado, Idaho, Montana, Nevada, New Mexico, Oregon, Utah, Washington, and Wyoming. Notes: * *p* < 0.05, ** *p* < 0.01, *** *p* < 0.001.

## Supplementary Materials

The following are available online at https://www.mdpi.com/1660-4601/16/6/1045/s1. **Table S1:** CDC Tracking Program questions and responses included in the 2013 SummerStyles survey. **Table S2:** Demographic characteristics for respondents, 2013 SummerStyles. **Table S3:** Univariate logistic regression: Demographic characteristics by awareness of governmental efforts on environmental health and concerns about health risks from environmental pollutants, United States, 2013.
