# Supplementary material for: Public Perceptions of Environmental Public Health Risks in the United States
Source: Int J Environ Res Public Health. 2019 Mar 22;16(6):1045. doi: 10.3390/ijerph16061045 (PMC6466406; doi:10.3390/ijerph16061045)
Supplement: Supplementary file 1 [file ijerph-16-01045-s001.pdf]

Supplementary Materials:

Table S1. CDC Tracking Program questions and responses included in the 2013 SummerStyles survey.

1) I am aware of the government's efforts to track environmental hazards and possible links to chronic health problems.<br>Strongly disagree Somewhat disagree Neither disagree nor agree Somewhat agree Strongly disagree
2) How concerned are you about the risks to your health from pollutants in the environment?<br>Not at all concerned Not very concerned Somewhat concerned Very concerned
3) What are the top 3 environmental issues you are concerned may impact YOUR health?<br>• Indoor air quality<br>• Lead in paint and consumer products<br>• Climate change<br>• Mercury in fish<br>• Outdoor air quality<br>• Electromagnetic field from electric and cell phone towers<br>• Pesticides<br>• None of these<br>• Chemicals in consumer products<br>• Drinking water quality<br>• Waste sites/landfills
4) Which of these do you think may be affected by the environment in YOUR community?<br>• Asthma<br>• Cancer<br>• Unhealthy pregnancies<br>• Carbon monoxide poisoning<br>• Respiratory illness<br>• Birth defects<br>• Childhood lead poisoning<br>• Unhealthy babies<br>• Heart attacks<br>• None of these
5) Where do you look for information on how the environment affects your health?<br>• Doctor or nurse<br>• Federal government<br>• Media (TV, magazines, newspapers)<br>• Other source<br>• Policymaker<br>• Health insurer<br>• Community organization<br>• Health department<br>• Non-profit organization<br>• I do not look for this information

Table S2. Demographic characteristics for respondents, 2013 SummerStyles[1]

Characteristic	*Unweighted n*	Weighted %
Total	4,033	100
Age		
18 - 34 years old	1,396	46.4
35 - 64 years old	1,770	35.3
65+ years old	867	18.3
Sex		
Male	2,113	51.8
Female	1,920	48.2
Race/ethnicity		
Non-Hispanic white	3,089	66.8
Non-Hispanic black	363	11.3
Hispanic	369	14.8
Non-Hispanic Other [2]	212	7.1
Education		
High school graduate or less	1,349	41.5

Some college	1,298	29.0
College graduate or higher	1,386	29.5
Marital status		
Married	2,371	53.5
Partnership	276	37.9
Other	1,386	8.6
Health status		
Excellent, very good	1,923	45.8
Good	1,514	38.9
Fair, poor	584	15.3
Employment status [3]		
Employed	2,287	54.5
Not employed	875	27.5
Retired	871	18.0
Household income		
< $34,900	1,025	26.9
$35,000 - $75,000	1,462	34.9
> $75,000	1,546	38.2
Household size		
1	687	14.0
2	1,512	34.5
3	761	19.9
4	624	17.3
5 more people	449	14.3
House ownership		
Yes	3,095	73.0
No	938	27.0
Region [4]		
Midwest	1,014	21.4
Northeast	722	18.3
South	1,408	37.6
West	889	22.7

[1] All data were weighted and the resulting numbers were rounded to the nearest whole number.

[2] Other race/ethnicity =Non-Hispanic other races or two more races.

[3] Employment status response of employed = "part- or full-time, self-employed", not employed = "temporarily unemployed, disabled, looking for job, not employed-other," and retired.

[4] Northeast includes: Connecticut, Maine, Massachusetts, New Hampshire, Rhode Island, Vermont, New Jersey, New York, and Pennsylvania. Midwest includes: Illinois, Indiana, Iowa, Kansas, Michigan, Minnesota, Missouri, Nebraska, North Dakota, Ohio, South Dakota, and Wisconsin. South includes: Alabama, Arkansas, Delaware, Florida, Georgia, Kentucky, Louisiana, Maryland, Mississippi, North Carolina, Oklahoma, South Carolina, Tennessee, Texas, Virginia, Washington, D.C., and West Virginia. West includes: Arizona, California, Colorado, Idaho, Montana, Nevada, New Mexico, Oregon, Utah, Washington, and Wyoming.

Table S3. Univariate logistic regression: Demographic characteristics by awareness of governmental efforts on environmental health and concerns about health risks from environmental pollutants, United States, 2013.

ConsumerStyles© characteristics	Awareness of the government's efforts to track environmental hazards and possible links to chronic health problems		Concern about the risks to health from pollutants in the environment
	Odds ratios (95% CI)		
	Aware vs. not aware [1]	Neutral vs. not aware [1]	Concerned vs. not concerned [2]
Race/ethnicity			
Non-Hispanic white	Ref	Ref	Ref
Non-Hispanic black	1.14 (0.72–1.81)	2.04 (1.30–3.21)**	1.69 (1.21–2.36)**
Hispanic	0.82 (0.55–1.25)	1.37 (0.92–2.03)	1.38 (1.02–1.88)*
Other [3]	0.87 (0.48–1.55)	1.21 (0.69–2.13)	1.89 (1.22–2.95)*
Age (years)			
18 - 34	Ref	Ref	Ref
35 - 64	1.27 (0.97–1.68)	1.05 (0.80–1.38)	1.14 (0.93–1.41)
65+	2.07 (1.47–2.90)***	1.21 (0.85–1.71)	0.90 (0.70–1.14)
Sex			
Male	1.50 (1.16–1.92)**	1.37 (1.06–1.77)*	Ref
Female	Ref	Ref	1.49 (1.23–1.79)***
Education			
High school graduate or less	Ref	Ref	Ref
Some college	1.40 (1.03–1.89)*	1.11 (0.82–1.49)	1.00 (0.80–1.25)
College graduate or higher	1.96 (1.45–2.65)***	1.07 (0.79–1.46)	1.24 (0.99–1.56)
Marital status			
Married	Ref	Ref	Ref
Partnership	1.05 (0.81–1.37)	0.96 (0.74-1.27)	0.95 (0.78–1.16)
Other	0.75 (0.45–1.23)	0.86 (0.52-1.40)	1.02 (0.70–1.48)
Health status			
Excellent, very good	1.52 (1.19–1.95)**	1.13 (0.88–1.46)	Ref
Good, fair, poor	Ref	Ref	1.26 (1.05–1.52)*
Employment status [4]			
Employed	Ref	Ref	Ref
Not employed	0.75(0.56–1.02)	1.09 (0.81–1.48)	1.17 (0.93–1.48)
Retired	1.76 (1.29-2.41)***	1.38 (0.99-1.91)	0.84 (0.67-1.06)
Household income			
< $34,900	Ref	Ref	Ref
$35,000 - $75,000	1.18 (0.86–1.64)	1.06 (0.77–1.46)	0.79 (0.62–0.99)
> $75,000	1.39 (1.01–1.90)*	0.91 (0.66–1.25)	0.97 (0.76–1.23)
Household size			
1 – 2 people	Ref	Ref	Ref
3 or more people	0.89 (0.69–1.13)	1.11 (0.87–1.43)	0.99 (0.82–1.19)
House ownership			
Yes	Ref	Ref	Ref
No	0.89 (0.66–1.19)	1.31 (0.98–1.76)	1.15 (0.93–1.44)
Region [5]			
Midwest	Ref	Ref	Ref
Northeast	0.95 (0.64–1.39)	1.08 (0.72–1.61)	1.30 (0.98–1.73)

South	0.86 (0.62–1.20)	1.16 (0.83–1.62)	1.35 (1.06–1.72)*
West	0.84 (0.58–1.21)	1.14 (0.78–1.66)	1.58 (1.20–2.08)**

[1] Aware = "strongly agree" and "somewhat agree" responses; not aware = "strongly disagree" and "somewhat disagree" responses; and neutral = "neither disagree nor agree" responses.

[2] Concerned = "very concerned" and "somewhat concerned" responses and not concerned = "not at all concerned" and "not very concerned" responses.

[3] Other race/ethnicity =Non-Hispanic other races or two more races.

[4] Employment status response of employed = "part- or full-time, self-employed", not employed = "temporarily unemployed, disabled, looking for job, not employed-other," and retired.

[5] Northeast includes: Connecticut, Maine, Massachusetts, New Hampshire, Rhode Island, Vermont, New Jersey, New York, and Pennsylvania. Midwest includes: Illinois, Indiana, Iowa, Kansas, Michigan, Minnesota, Missouri, Nebraska, North Dakota, Ohio, South Dakota, and Wisconsin. South includes: Alabama, Arkansas, Delaware, Florida, Georgia, Kentucky, Louisiana, Maryland, Mississippi, North Carolina, Oklahoma, South Carolina, Tennessee, Texas, Virginia, Washington, D.C., and West Virginia. West includes: Arizona, California, Colorado, Idaho, Montana, Nevada, New Mexico, Oregon, Utah, Washington, and Wyoming. Notes: * $p < 0.05$, ** $p < 0.01$, *** $p < 0.001$.
